# Group A *Streptococcus* induces lysosomal dysfunction in THP-1 macrophages

**DOI:** 10.1128/iai.00141-24

**Published:** 2024-05-09

**Authors:** Scott T. Nishioka, Joshua Snipper, Jimin Lee, Joshua Schapiro, Robert Z. Zhang, Hyewon Abe, Andreas Till, Cheryl Y. M. Okumura

**Affiliations:** 1 Biology Department, Occidental College, Los Angeles, California, USA; 2 Division of Biological Sciences, University of California San Diego, La Jolla, California, USA; 3 The San Diego Center for Systems Biology, University of California San Diego, La Jolla, California, USA; 4 University Hospital of Bonn, Bonn, Germany; Universite de Geneve, Geneva, Switzerland

**Keywords:** group A *Streptococcus*, streptolysin O (SLO), macrophage, lysosome, CD63, cathepsin B

## Abstract

The human-specific bacterial pathogen group A *Streptococcus* (GAS) is a significant cause of morbidity and mortality. Macrophages are important to control GAS infection, but previous data indicate that GAS can persist in macrophages. In this study, we detail the molecular mechanisms by which GAS survives in THP-1 macrophages. Our fluorescence microscopy studies demonstrate that GAS is readily phagocytosed by macrophages, but persists within phagolysosomes. These phagolysosomes are not acidified, which is in agreement with our findings that GAS cannot survive in low pH environments. We find that the secreted pore-forming toxin Streptolysin O (SLO) perforates the phagolysosomal membrane, allowing leakage of not only protons but also large proteins including the lysosomal protease cathepsin B. Additionally, GAS recruits CD63/LAMP-3, which may contribute to lysosomal permeabilization, especially in the absence of SLO. Thus, although GAS does not inhibit fusion of the lysosome with the phagosome, it has multiple mechanisms to prevent proper phagolysosome function, allowing for persistence of the bacteria within the macrophage. This has important implications for not only the initial response but also the overall functionality of the macrophages, which may lead to the resulting pathologies in GAS infection. Our data suggest that therapies aimed at improving macrophage function may positively impact patient outcomes in GAS infection.

## INTRODUCTION

Group A *Streptococcus* (GAS) is a significant cause of morbidity and mortality worldwide, especially in medically underserved areas where antibiotic therapy may not be promptly available. Infection produces wide-ranging clinical manifestations including pharyngitis, acute rheumatic fever (ARF) often leading to rheumatic heart disease, and invasive disease, including necrotizing fasciitis (1). Acting as the first line of defense against such infections, macrophages are critical for the early control and resolution of GAS infection (2, 3). However, GAS has been shown to survive intracellularly in macrophages during both acute invasive soft tissue infection and asymptomatic carriage (3–5). Although GAS remains highly sensitive to beta-lactam antibiotics, the gold standard of treatment (6), the ability of bacteria to persist in macrophages and re-emerge after antibiotic treatment poses a therapeutic challenge (4).

As both phagocytes and antigen-presenting cells, macrophages function at the intersection of innate and adaptive immunity and are therefore critical for identifying pathogens and defending against infection. Macrophages engulf bacterial pathogens into phagosomes, which fuse with lysosomes that deliver proteolytic enzymes to facilitate bacterial destruction. These enzymes have optimal proteolytic activity under acidic conditions, and therefore lysosomal acidification is important for macrophage bactericidal function and subsequent antigen presentation (7). Inhibition of either lysosomal fusion with bacteria-containing phagosomes or phagolysosomal acidification is an evasion tactic employed by several bacterial pathogens including *Mycobacterium tuberculosis*, *Legionella pneumophila*, and *Staphylococcus aureus* (7–9). GAS may employ similar tactics to survive intracellularly within macrophages and to evade the adaptive immune response upon host re-infection, which may contribute to the development of pathologies such as toxic shock syndrome (10).

GAS is a human-specific pathogen and has evolved mechanisms to survive the immune response (11). Previous data have indicated that phagocytosed GAS can prevent fusion with destructive organelles such as azurophilic granules and lysosomes (12, 13). Other reports have shown that although GAS-containing phagosomes do fuse with lysosomes, GAS survives within macrophages via the secretion of virulence factors such as Streptolysin O (SLO) and NADase (14, 15). SLO is a cholesterol-dependent cytolysin that oligomerizes to form large pores (~30 nm) within the host cell membrane (16) that allows the loss of proteins up to 15 nm in diameter (17). As a result, SLO is hypothesized to permeabilize the phagolysosomal membrane, permitting free flow of protons out of the phagolysosome and limiting the activity of lysosomal proteases that facilitate intracellular killing (15, 18). Interestingly, it has also been reported that GAS is capable of preventing lysosomal acidification by preventing the recruitment of the vacuolar ATPase (v-ATPase) (19). In the context of these data, it is unclear what mechanisms are contributing to GAS intracellular survival in macrophages. In this paper, we used the human monocytic cell line THP-1 differentiated into macrophages to clarify the molecular mechanism by which GAS survives in macrophages. We find that not only does GAS induce leakage of large proteins from the phagolysosome into the cytosol, but that acidification of the phagolysosome is limited even in strains lacking SLO. This has important consequences for the survival of the bacteria, requiring macrophages to mount a second-line defense, and underscores the need to elucidate these events to improve patient outcomes.

## MATERIALS AND METHODS

### Antibodies and chemicals

Antibodies to the following proteins were used in this study: EEA-1 (Abcam, ab2900), LAMP-2 (Abcam, ab25631), V_0_D_1_ (Abcam, ab56441), cathepsin B (Cell Signaling Technology, D1C7Y), LAMP-1 (Cell Signaling Technology, #9091), V_1_A (Abnova, H00000523-A01), beta-actin (Thermo Fisher Scientific, MA5-15739), and CD63 (Developmental Studies Hybridoma Bank, H5C6). Fluorescent secondary antibodies were purchased from Thermo Fisher Scientific. The following fluorescent probes were purchased from Thermo Fisher Scientific: Alexa Fluor 488 Hydrazide (A10436), Oregon Green 488 Anionic, Lysine Fixable Dextran 10,000 MW (D7171) and 70,000 MW (D7173), and LysoTracker DeepRed (L12492). Fluorescein isothiocyanate (FITC) dextrans (40,000 and 70,000 MW) were purchased from MilliporeSigma. To generate antibody-coupled beads, 0.1-μm Carboxylate-Modified Microspheres (Thermo Fisher Scientific) were coupled to approximately 400-mg normal pooled human serum (Thermo Fisher Scientific) using 2% 1-ethyl-3-(3-dimethylaminopropyl) carbodiimide hydrochloride (QBiosciences). The reaction was quenched with 40-mM ethanolamine and resuspended in phosphate-buffered saline (PBS). Successful coupling of the beads was assessed by fluorescence microscopy showing increased phagocytosis by THP-1 macrophages compared with uncoupled beads (data not shown). One millimolar L-leucyl L-leucine O-methyl ester (LLOMe, Cayman Chemical, #16008) was incubated with cells to induce lysosomal damage.

### Bacterial strains

Wild-type (WT) GAS strain M1T1 5448 (M1 GAS) was originally isolated from a patient with necrotizing fasciitis and toxic shock syndrome (20). Isogenic mutant strains lacking Streptolysin O (ΔSLO), Streptolysin S (via lack of SagA, ΔSagA), and Emm1 (ΔM1) were previously described (21–23). Bacterial strains were cultivated in Todd-Hewitt broth (THB) at 37°C. For all experiments, bacteria were grown to log phase in the presence of 1:200 pooled normal human serum (Thermo Fisher Scientific) to opsonize bacteria. The gene for monomeric WASABI (mWASABI) (24, 25) was codon-optimized for expression in bacteria and was synthesized in a shuttle vector (GenScript; see supplemental data for amino acid sequence). The gene was subcloned into pDCerm (26), and the resulting plasmid (pWASABI) was transformed into WT M1 GAS and the ΔSLO mutant. Plasmid-bearing strains were maintained in THB supplemented with 5 μg/mL erythromycin. Heat-killed (HK) bacteria were prepared by incubating a known concentration of bacteria at 95°C for 10 min, followed by a 15-min opsonization in 1:200 pooled normal human serum at room temperature.

### Cell culture

THP-1 cells were purchased from Sigma (cat. 88081201) and were cultured in RPMI supplemented with 10% heat-inactivated fetal bovine serum (FBS) (Corning), 2-mM L-glutamine, and 100 U/mL penicillin/100 µg/mL streptomycin at 37°C/5% CO_2_. Cells were differentiated to macrophages using 20-nM phorbol 12-myristate 13-acetate (PMA) (MilliporeSigma) 24–48 h prior to experiments.

### Immunofluorescence

THP-1 cells (2.5 × 10^5^) were seeded on coverslips in the presence of 20-nM PMA 24–48 h prior to experiments. For dextran leakage assays, cells were incubated in media containing 20 μg/mL of the indicated dextran or 500 μg/mL 70-kD dextran overnight, washed and chased in cell culture media for 2 h prior to bacterial infection. For infection experiments with two rounds of infection, secondary infection bacteria were labeled with 10 μM CellTracker Orange (Thermo Fisher Scientific) 30 min prior to infection. Bacteria were combined with cells at an multiplicity of infection (MOI) = 10 in RPMI supplemented with 2% FBS only (no antibiotics), and plates were centrifuged at 500 × *g* to synchronize bacterial contact with cells. At the indicated time points, cells were fixed with 4% paraformaldehyde or 3.7% formaldehyde for 10 min at room temperature. Cells were incubated with blocking solution [10% goat serum, 3% bovine serum albumin (BSA), and 0.1% Triton X-100 in PBS], then incubated with the indicated primary antibodies for 1 h at room temperature in block solution. Cells were washed and incubated with the indicated secondary antibodies for 1 h at room temperature. GAS opsonized in human serum was detected using fluorescent anti-human IgG antibodies where indicated. Cells were washed and mounted onto slides with ProLong antifade reagent with DAPI (Thermo Fisher Scientific). Slides were imaged on an inverted Leica TCS SP5 confocal microscope using a 63x/1.40 oil objective with 2–3x digital zoom at calibrated magnifications and were recorded with LAS AF software (Leica). Quantitation of dextran, LAMP-1, and CD63 colocalization with bacteria was analyzed with CellProfiler 4.2.6 (27) using the Otsu thresholding method and the Colocalization pipeline. To automatically count bacteria and cells, either fluorescence (bacteria) or brightfield (THP-1 macrophages) images were thresholded, objects were filled, and average object size was used to count cells. For LAMP-1 and CD63, colocalization is expressed as the percentage of GAS fluorescence that colocalized with LAMP-1 or CD63 fluorescence, and the number of bacteria per cell was estimated by dividing the total number of bacteria by the total number of cells counted in each image. For dextran staining, colocalization is expressed as the percentage of dextran fluorescence that colocalized with GAS or CD63 fluorescence. For quantitation of mWASABI-expressing bacteria within phagosomes, images were coded and counted manually in a blind fashion by at least three independent reviewers. For manually counted cells, the number of bacteria per cell and the number of infected cells were calculated by averaging the number of bacteria in infected cells across all counted images within an experiment. For all imaging data, at least three independent experiments were performed, at least 16 images containing >10 cells per image were analyzed, and >100 cells were counted per time point.

### Verification of pH sensitivity of mWASABI in GAS

To assess the fluorescence intensity of mWASABI in live GAS, log phase cultures of bacteria were incubated on coverslips in THB with 10 mM HEPES adjusted to the indicated pH for 1 h at 37°C. Bacteria were fixed with 2% paraformaldehyde and were analyzed by confocal microscopy. Gain settings were adjusted to bacteria incubated in pH 7 media, and the settings were applied to all other conditions. Images were thresholded, and the corrected total cell fluorescence (integrated density – average background integrated density) of each bacterial cell was measured using ImageJ.

### Acidification assay

THP-1 cells (10^5^) were seeded with 20-nM PMA in 96-well black plates with clear bottoms overnight. The following day, cells were fed 500 μg/mL 70-kD FITC dextran overnight. On the third day, cells were chased with normal cell culture media (RPM1 supplemented with 10% FBS, no antibiotics) for 2 h prior to infection. Cells were infected at an MOI of 10 with either the indicated bacterial strain or Ab-conjugated latex beads in RPMI supplemented with 2% FBS only (no antibiotics), and plates were centrifuged at 500 × *g* to synchronize bacterial contact with cells. Fluorescence intensity at 480 nm/535 nm (Em/Ex) was monitored at 37°C in a fluorescent plate reader for 3 h.

### Bacterial survival in different pH media

Bacteria were incubated in THB with 10 mM HEPES adjusted to the indicated pH. For bacterial growth, cultures were adjusted to an OD_600nm_ of 0.1 and were incubated at 37°C. OD_600nm_ was measured every 15 min. For bacterial survival, 2 × 10^5^ CFU log phase bacteria were added to 250-μL pH-adjusted culture media in 96-well plates. At the indicated time point, a 25-μL aliquot was removed from the culture and was quantitated by plating on agar plates.

### Cell fractionation and Western blot

Following a 30-min infection period, a total of 10^7^ PMA-differentiated cells were scraped and collected in 1 mL of fractionation buffer (50 mM KCl, 90 mM K-Gluconate, 1 mM EGTA, 5 mM MgCl2, 50 mM sucrose, 20 mM HEPES, pH 7.4, 5 mM glucose, 1X HALT phosphatase/protease inhibitor cocktail [Thermo Fisher Scientific], 1 μg/mL pepstatin [MilliporeSigma], and 1 mM PMSF [MilliporeSigma]). Cells were lysed by nitrogen cavitation equilibrated on ice for 30 min at 400 psi. The resulting lysates were fractionated into membrane and cytosolic fractions by centrifugation at 16,000 × *g* at 4°C for 15 min (pellet = membrane fraction, supernatant = cytosolic fraction). Protein concentrations were measured by BCA assay (Thermo Fisher Scientific). Each sample (10 μg) was run on SDS-PAGE gels and was transferred to a 0.2-μm polyvinylidene difluoride (PVDF) membrane. Blots were blocked with 5% (wt/vol) non-fat dry milk in 1X Tris-buffered saline with 0.1% Tween 20 (TBST). Blots were probed with the indicated antibodies overnight at 4°C. Relative protein concentrations were quantified by densitometry analysis in ImageLab v. 6.0.1 (BioRad). v-ATPase assembly was measured by normalizing V_1_A densitometry values in the membrane fraction to the V_0_D_1_ loading controls in membrane fractions and by calculating the ratio of V_1_A in the sample compared with uninfected macrophages (28).

### Cathepsin B activity assay

PMA-differentiated THP-1 cells (4 × 10^6^) were infected at an MOI = 10 for 60 min, followed by lysis with 30 μg/mL digitonin in acetate buffer (50 mM Na-acetate, pH 5.6, 150 mM NaCl, 0.5 mM EDTA) and protease inhibitors (1X HALT phosphatase/protease inhibitor cocktail [Thermo Fisher Scientific], 100 µM PMSF, 1 µg/mL pepstatin) (29). The cell lysate was separated into cytosolic and membrane fractions as described above. Cathepsin B activity in cytosolic fractions was measured using the SensoLyte 520 Cathepsin B Assay Kit (AnaSpec) according to the manufacturer’s instructions. Relative fluorescence units were normalized to the corresponding protein concentration for each sample and were compared with the uninfected cells. Data from three independent experiments were combined.

### v-ATPase activity assay

Cell membrane fractions containing lysosomes were prepared using 30 μg/mL digitonin in acetate buffer as described above. Each sample (10 μg) was equilibrated in 200-μL assay buffer (10 mM HEPES, 5 mM MgCl2, 125 mM KCl, pH = 7.0) (30) at room temperature for 30 min in the presence or absence of 2-μM bafilomycin (MilliporeSigma) or 200-μM sodium orthovanadate (MilliporeSigma). Fresh ATP (1 mM) in 200 mM Tris pH = 7 was added to each sample and was incubated at room temperature for 2.5 h. Twenty-five microliters of the reaction was collected and diluted in ultrapure water, and liberation of free phosphate was measured using the Malachite Green Phosphate Assay Kit according to the manufacturer’s instructions (MilliporeSigma). Corrected absorbance values (= A_sample_ – A_substrate blank_ – A_buffer blank_) were compared with a phosphate standard curve to determine phosphate concentration. v-ATPase activity was calculated as the difference in phosphate concentration between samples incubated with and without bafilomycin. Na^+^/K^+^-ATPase activity was calculated as the difference in phosphate concentration between samples incubated with bafilomycin and with or without sodium orthovanadate.

### Intracellular bacteria recovery assay

PMA-differentiated THP-1 cells (4 × 10^6^) were infected at an MOI = 10 for 30 min, followed by treatment with 100 μg/mL gentamycin for 1 h to kill extracellular bacteria. After extensive washing, cells were lysed with 30 μg/mL digitonin in acetate buffer supplemented with protease inhibitors as described above. Cells were separated into cytosolic and bacteria/membrane fractions by centrifugation. Membrane fractions were further treated with 0.025% Triton X-100 to release bacteria from membrane-bound vesicles. All fractions were enumerated and compared with the total number of intracellular bacteria. Bacteria in fractions were stained with LAMP-1, CD63, or anti-human IgG antibodies to detect opsonized GAS as indicated. Image analysis indicated successful lysis of cells and liberation of bacteria (data not shown).

### Statistical analysis

Growth curve data were analyzed using the Growthcurver package in R (31), and comparison of the intrinsic growth rate (r) and carrying capacity (K) was analyzed by one-way analysis of variance (ANOVA) and Tukey’s post-hoc test. All other data were analyzed using Prism v. 10.2.0 (GraphPad Software), on arcsin-transformed data where indicated, by one-way ANOVA with Tukey’s (normally distributed data) or Dunnett’s or Kruskal-Wallis (nonparametric data) multiple comparison tests. Data in Fig. 2D and G; Fig. S4B were also analyzed by two-way ANOVA to ensure day-to-day experimentation and/or individual counters did not contribute to the differences observed in the data (data not shown). Outliers were assessed by the ROUT method (Q = 1%). For all data presented, ****P < 0.0001, ***P < 0.001, *P < 0.05, n.s., not significant.

## RESULTS

### Bacterial trafficking in THP-1 macrophages

**Fig 1:**
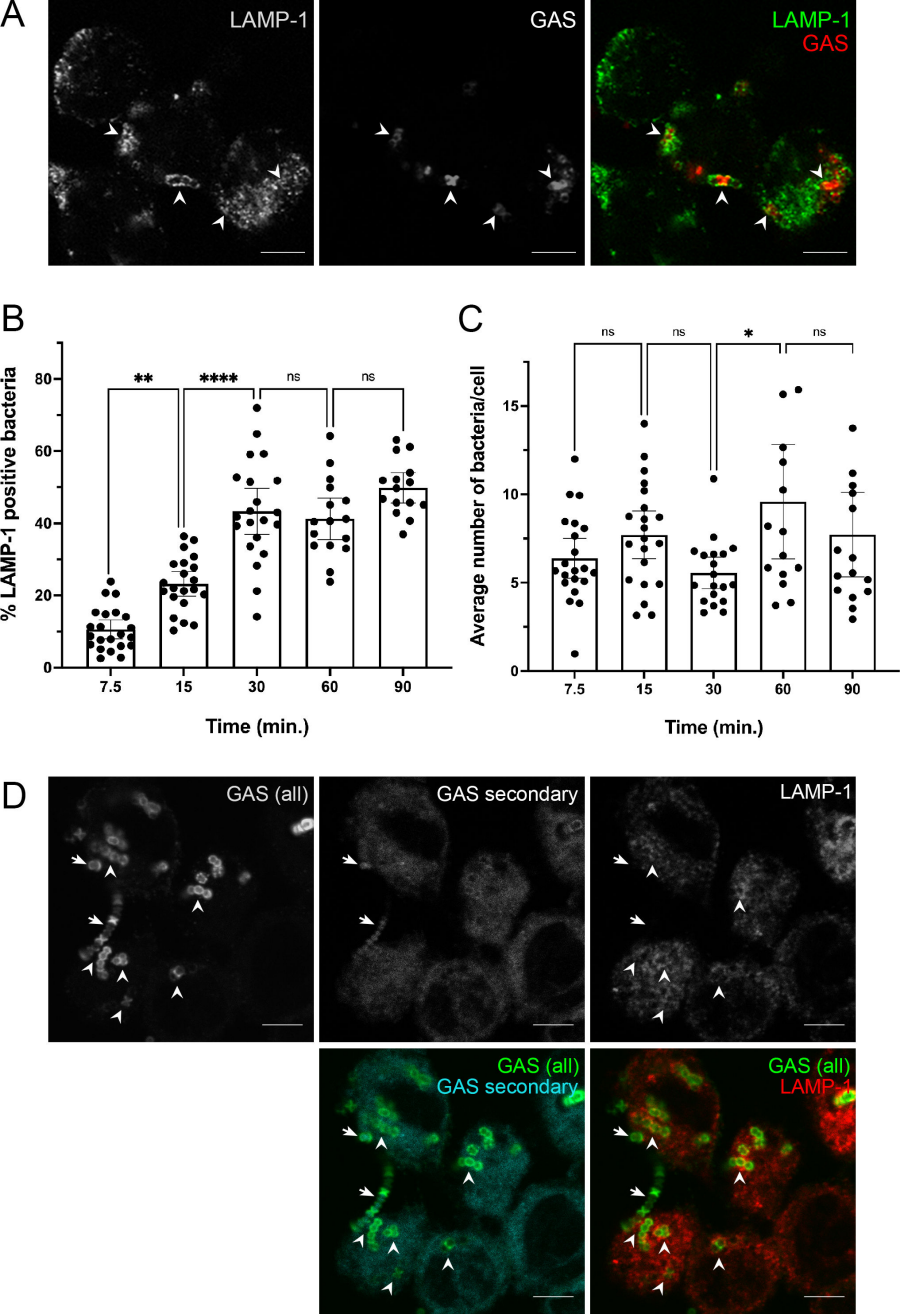
GAS persists in phagolysosomes of THP-1 cells. (**A, B**) THP-1 cells were infected with GAS for the indicated times, fixed and probed with anti-LAMP-1 and secondary fluorescent antibodies (green). Bacteria opsonized with human serum were detected with anti-human IgG antibody (red). (**A**) Representative image of LAMP-1 colocalized with GAS in THP-1 cells at 60 min post-infection. Arrowheads indicate examples of colocalization. Scale bar = 5 mm. (**B**) Quantitation of LAMP-1-positive bacteria at the indicated time points. (**C**) Average number of bacteria per cell at the indicated time points. (**D**) Representative image of THP-1 cells exposed to a primary (green only) GAS infection for 30 min, followed by a secondary (cyan and green) infection for 30 min (60-min total infection time). LAMP-1 staining is indicated in red. Arrowheads indicate examples of colocalization between primary-infecting GAS and LAMP-1. Arrows indicate GAS from the secondary infection. Scale bar = 5 mm. For all graphs, >100 cells were counted. Data from three independent experiments were combined. Results are given as mean ± 95% confidence interval (CI). Data were analyzed on arcsin-transformed data by one-way ANOVA with Tukey’s multiple comparison test.

**Fig 2:**
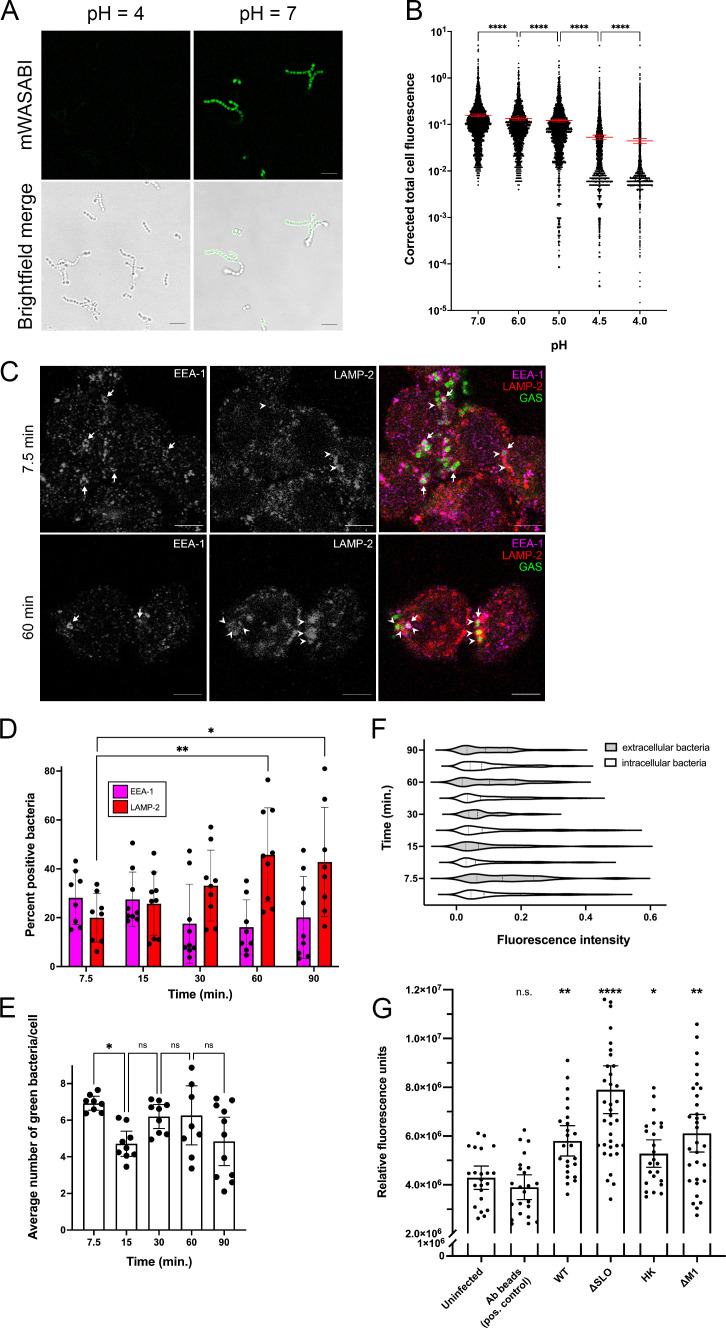
GAS persists in non-acidified phagolysosomes of THP-1 cells. (**A**) GAS expressing mWASABI was incubated in media at the indicated pH for 1 h. Representative green fluorescence (mWASABI) and brightfield images are shown. Scale bar = 5 mm. (**B**) Corrected total cell fluorescence of green fluorescence signal from bacteria expressing mWASABI incubated in media at the indicated pH for 1 h. (**C**) Representative fluorescence microscopy images of GAS-infected THP-1 cells at 7.5 and 60 min post-infection. Arrows denote examples of bacteria encapsulated in early phagosomes (EEA-1, magenta), and arrowheads denote examples of bacteria encapsulated in phagolysosomes (LAMP-2, red). All images were taken with a 63x objective with 2x digital zoom, scale bar = 5 mm. (**D**) Quantitation of bacteria colocalized with phagosomes (EEA-1) or phagolysosomes (LAMP-2) at the indicated time points. (**E**) Average number of bacteria per cell at the indicated time points. (**F**) Violin plot of fluorescence signal intensity from intracellular (clear bars) or extracellular (shaded bars) bacteria expressing mWASABI. (**G**) Fluorescence signal from cells fed 70-kD FITC dextran and infected with immunoglobulin-conjugated beads (Ab beads); the indicated GAS strain or HK bacteria were measured by plate reader. Statistical differences were calculated by comparing samples to uninfected cells. Data from at least three independent experiments were combined. For (**D**) and (**E**), >100 cells were counted. Data from at least three individual counters of at least three independent experiments are shown. Results are given as mean ± 95% confidence interval (CI), and statistics were performed on arcsin-transformed data by one-way ANOVA with Dunnett’s multiple comparison test.

Because there are reports that GAS can prevent (13) or allow (15) lysosomal fusion upon phagocytosis, we first examined the intracellular trafficking of M1 5448 GAS in THP-1 macrophages. Bacterial contact with cells was initiated by centrifugation to synchronize macrophage uptake of bacteria, and phagolysosomal maturation was monitored by immunofluorescence in a short time course infection experiment (Fig. 1 and 2). In agreement with the literature (13, 15, 32), WT bacteria were rapidly engulfed by THP-1 cells by our earliest measured time point at 7.5 min (Fig. 1B and 2D) and were colocalized with the early phagosomal marker EEA-1 (Fig. 2C and D). Interestingly, bacteria in phagosomes also began colocalizing with the lysosomal marker LAMP-1 as early as 7.5 min (Fig. 1B). Maximal colocalization with LAMP-1 began at 30 min, and GAS remained within phagolysosomes throughout the later time points (Fig. 1A and B). Surprisingly, but in agreement with other reports (32), the number of bacteria per cell was relatively constant throughout the experiment (Fig. 1C). These data indicated that bacteria were trafficked as expected to phagolysosomes, but bacteria were held in these compartments without observable bacterial elimination or replication within this time frame. Although we synchronized the infection, there was a possibility that this result was due to continuous infection of macrophages or bacterial lysis of macrophages and re-infection. To address this, we performed the trafficking experiment with a second round of bacterial infection (Fig. 1D). Bacteria introduced in the secondary infection were rarely found in macrophages, were mostly extracellular, and did not colocalize with LAMP-1 (Fig. 1D), in agreement with a report for *Salmonella* Typhimurium that the frequency of macrophage reinfection after the primary infection is rare (33). Supporting this data, the percentage of infected cells was similar across all time points (Fig. S1). Thus, bacteria from the primary infection persist in THP-1 macrophage phagolysosomes.

### GAS resides in non-acidified phagolysosomes in THP-1 macrophages

Acidification is critical for optimal activity of the proteolytic enzymes in the lysosome (34). Because bacteria appeared to be held in phagolysosomes without observable elimination, we wondered whether phagolysosomes containing GAS were acidified. Lysotracker is an acidotropic dye commonly used to track acidic compartments such as lysosomes in the cell and has been used in previous work to determine acidification of GAS-infected phagolysosomes (15, 32). Although Lysotracker colocalized with LAMP-1 in THP-1 cells, the staining and uptake were inconsistent (Fig. S2A). Furthermore, unlike the staining pattern we saw with LAMP-1, we noted that we could not easily observe endo- and lysosomal staining with Lysotracker in our infected cells and that Lysotracker mostly stained bacteria (Fig. S2A). Because GAS is an acid-producing bacterial species, we tested whether Lysotracker directly stained GAS. WT bacteria were brightly stained with Lysotracker both in infected cells and in the absence of cells (Fig. S2A through C), which made it difficult to determine whether and to what extent phagolysosomes containing GAS were acidified. This raised questions about whether the previous work accurately measured GAS in acidified phagolysosomes, because Lysotracker was added to cells after GAS infection and could stain bacteria due to lactic acid production, and lysosomal staining was otherwise not observed (15). We therefore developed an alternative method to measure intralysosomal pH by expressing a pH-sensitive green fluorescent protein variant mWASABI (25) in GAS to monitor phagolysosomal pH of live GAS in cells (Fig. 2A). Green fluorescence produced by live bacteria was appropriately quenched in low pH environments (Fig. 2A and B). Notably, pH was distinguishable by fluorescence intensity between pH 5.0 and 4.5 (Fig. 2B).

We infected THP-1 macrophages with mWASABI-expressing GAS and performed a detailed trafficking assay of GAS through the phagolysosomal pathway (Fig. 2C and D). For this experiment, we used the lysosomal protein LAMP-2 to confirm the lysosomal fusion we observed earlier (Fig. 1) because there is conflicting evidence in the literature that GAS prevents lysosomal fusion (13). Because bacteria were tracked by mWASABI expression instead of immunofluorescent staining, colocalization with phagosomal markers was difficult with automated methods (Fig. 2C and D). To quantitate the data, we manually counted bacteria that colocalized with phagolysosomal markers in a blinded manner (Fig. 2D). We again found that GAS was initially located in phagosomes, as indicated by EEA-1 staining, which was followed by rapid bacterial colocalization with the mature lysosomal marker LAMP-2 within the first 15 min of infection (Fig. 2C and D), similar to the colocalization dynamics observed with LAMP-1 staining (Fig. 1). An average of 50% of the counted intracellular bacteria were colocalized with LAMP-2 at the 60 and 90 min post-infection time points, which was significantly increased compared with the 7.5 min time point (Fig. 2C and D). Although there were some fluctuations in the number of bacteria in phagolysosomes (Fig. 2E), the number of intracellular bacteria remained relatively constant at all time points, consistent with our initial data (Fig. 1C) and other reports (32). This corroborated our finding that little to no bacterial eradication or replication was occurring within this time frame (Fig. 2E).

We also found that the green fluorescence signal did not diminish over time, indicating that the intracellular bacteria were not in an acidified environment (Fig. 2C and F), in agreement with previous work that used Lysotracker (15, 32). Fluorescence signal intensity of intracellular bacteria in phagolysosomes was not significantly different than that of the extracellular bacteria in our images (Fig. 2F). As the lysosomal lumen is typically between pH 4.5 and 5 when acidified (35), a pH level that our mWASABI probe can differentiate (Fig. 2A and B), these data confirmed that GAS was maintained in phagolysosomes that are not appropriately acidified.

Although able to discern a pH drop to 4.5 or lower *in vitro*, our mWASABI probe still shows variable fluorescence signal (Fig. 2B). Furthermore, the pH of lysosomes could be lowered to 5, which our probe would have difficulty differentiating in THP-1 cells (Fig. 2B). We therefore confirmed that GAS-infected phagosomes were not acidified with a second method using pH-sensitive FITC-conjugated 70-kD dextran particles. Acidification of the lysosomes resulted in quenching of the fluorescence signal in uninfected and control cells infected with immunoglobulin-conjugated beads (Fig. 2G). However, the fluorescence signal was increased in cells infected with all tested strains of GAS, indicating little to no pH drop (Fig. 2G). The low magnitude of difference we observed in our GAS-infected cells compared with the uninfected cells may be due to the limited ability of cells to uptake such a large molecule (70 kD), which produced variation between experiments (Fig. 2G). Nevertheless, these results confirmed that GAS-infected phagolysosomes are not properly acidified. Although there are differences in the dextran fluorescence signal between GAS strains (Fig. 2G), all cells infected with GAS show increased fluorescence signal compared with the uninfected cells. This ability of GAS to prevent lysosomal acidification may account for the persistence of the bacteria in THP-1 cells (Fig. 1C and 2E).

### GAS does not tolerate acidic lysosomal conditions

**Fig 3:**
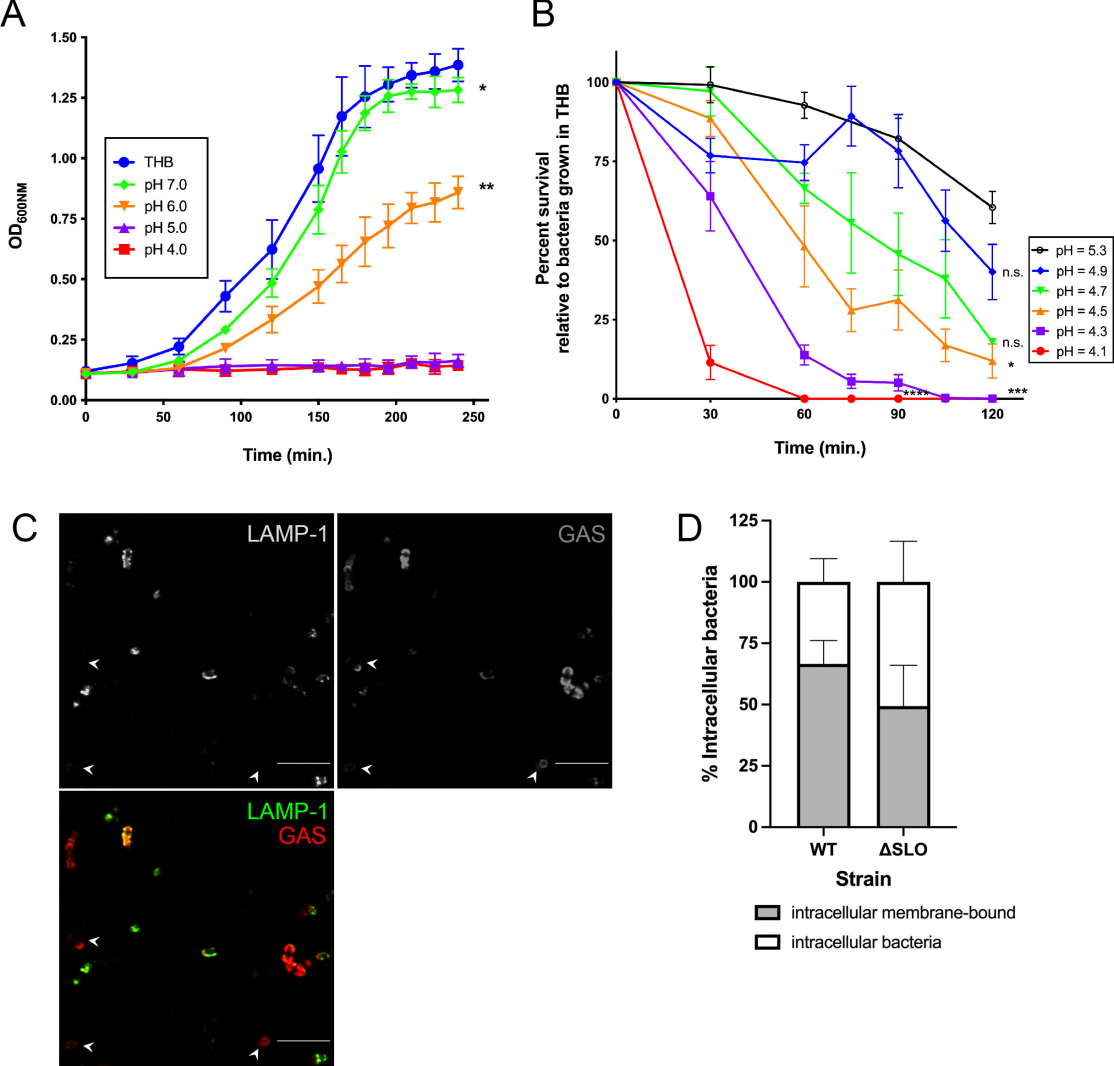
GAS does not survive in low pH environments. (**A**) Growth curves of bacteria grown in buffered media or unbuffered bacterial media (THB) at the indicated pH. Results are given as mean ± 95% confidence interval (CI). Statistics in (**A**) indicate differences in intrinsic growth rate compared with bacteria grown in unbuffered media tested by one-way ANOVA with Tukey’s multiple comparison test. (**B**) Bacterial survival in buffered media at the indicated pH compared with survival of bacteria in unbuffered bacterial media (THB). Statistics in (**B**) indicate differences in area under the curve compared with bacteria grown in pH = 5.3 media tested by one-way ANOVA with Tukey’s multiple comparison test. For each experiment, samples were prepared in triplicate. Data from at least three independent experiments were combined. Results are given as mean ± SEM. (**C**) Intracellular bacteria were recovered and probed with anti-LAMP-1 and secondary fluorescent antibodies (green). Bacteria opsonized with human serum were detected with anti-human IgG antibody (red). Arrowheads indicate examples of bacteria that were not in LAMP-1 vesicles. Scale bar = 7.5 mm. (**D**) Enumeration of total recovered (open bar) and membrane-bound (shaded bar) intracellular bacteria. No statistically significant differences between strains were found. Data from three independent experiments were combined, and results are given as mean ± 95% CI.

GAS is a lactic acid-producing bacterial species and as expected, we found that the final pH of both buffered and unbuffered culture medium after bacterial growth was acidic (Table 1). To cope with pH changes in both the lysosome and the environment, GAS possesses systems, such as the arginine deiminase (ADI) pathway, which confer acid tolerance (36, 37). However, in a physically constrained environment such as the lysosome, the effectiveness of such systems may be limited. The pH of the lysosomal lumen has been reported to range between pH 4.5 and 5. We wondered whether a pH similar to that found in the lysosome adversely affected bacterial growth and survival. In timed growth experiments, bacteria grown in buffered media adjusted to pH 7 had no significant difference in maximum population size but a statistically significant increase in the intrinsic growth rate of the population compared with unbuffered media (Fig. 3A). Bacteria grown in media adjusted to pH 6 grew at a significantly slower rate and did not reach a similar maximum population size in the time frame of our experiment compared with unbuffered media (Fig. 3A). Bacteria grown in media adjusted to pH 5 or lower did not demonstrate measurable growth (Fig. 3A). Thus, GAS growth is sensitive to decreasing pH and is inhibited at a pH found in the lysosomal environment.

**TABLE 1:** Final pH of media after bacterial growth, mean ± SD

Starting pH	4.0	5.0	6.0	7.0	Unbuffered THB (6.6)
Ending pH	4.10 ± 0.02	5.07 ± 0.02	4.99 ± 0.01	5.23 ± 0.08	5.63 ± 0.06

The final pH of the stationary phase cultures in both buffered and unbuffered culture media (Table 1) indicated that although GAS may not replicate in acidic conditions, it may be capable of survival. Quantitative plating of cultures at various time points throughout the growth experiment indicated that bacteria in pH 5 media were present (data not shown) but not measurably growing (Fig. 3A). We therefore performed a detailed time course to monitor bacterial survival in low pH media (Fig. 3B). Bacteria incubated in unbuffered media did not significantly grow during the time frame of the survival experiment (data not shown). Similar to other studies (38, 39), we found that GAS does not survive in low pH media (Fig. 3B). Although bacteria incubated in up to pH 5.3 media could survive for a short time, bacterial numbers declined at later time points (Fig. 3B), in agreement with pH 5 being the limit at which bacteria can survive (37, 40) and the final pH of stationary phase bacterial cultures (Table 1).

In our previous experiment, loss of fluorescence of mWASABI measurably occurs between pH 4.5 and 5, where bacteria at pH 5 largely remain fluorescent (Fig. 2B). It is therefore feasible that phagolysosomes could reach a lethal pH of 5 (Fig. 3B) that we could not detect with our mWASABI probe. In order to confirm that GAS in phagolysosomes was viable, we infected cells followed by treatment with antibiotics to kill extracellular bacteria and ensure recovery of only intracellular bacteria. We lysed the plasma membrane with digitonin and recovered intracellular bacteria both in LAMP-1-positive vesicles as well as unstained GAS (Fig. 3C), suggesting bacterial escape to the cytosol as reported in other work (32). We then liberated membrane-bound bacteria using Triton and found that membrane-bound bacteria were viable and accounted for over 50% of the recovered intracellular bacteria (Fig. 3D). These data are in line with our previous data showing that approximately 50% of bacteria are in phagolysosomes (Fig. 1B and 2D) and provide further support that these phagolysosomes are not acidified. We also tested cytotoxicity of GAS to THP-1 cells within the time frame of our experiments and found no significant cytotoxicity (Fig. S3), demonstrating that we recovered GAS from live cells. These data affirm that GAS cannot survive in an acidified lysosome (Fig. 3B) and therefore, limiting or preventing acidification (Fig. 2 and 3) is a survival strategy that GAS may use to persist in phagolysosomes in live macrophages.

### Streptolysin O creates large perforations in the phagolysosome

**Fig 4:**
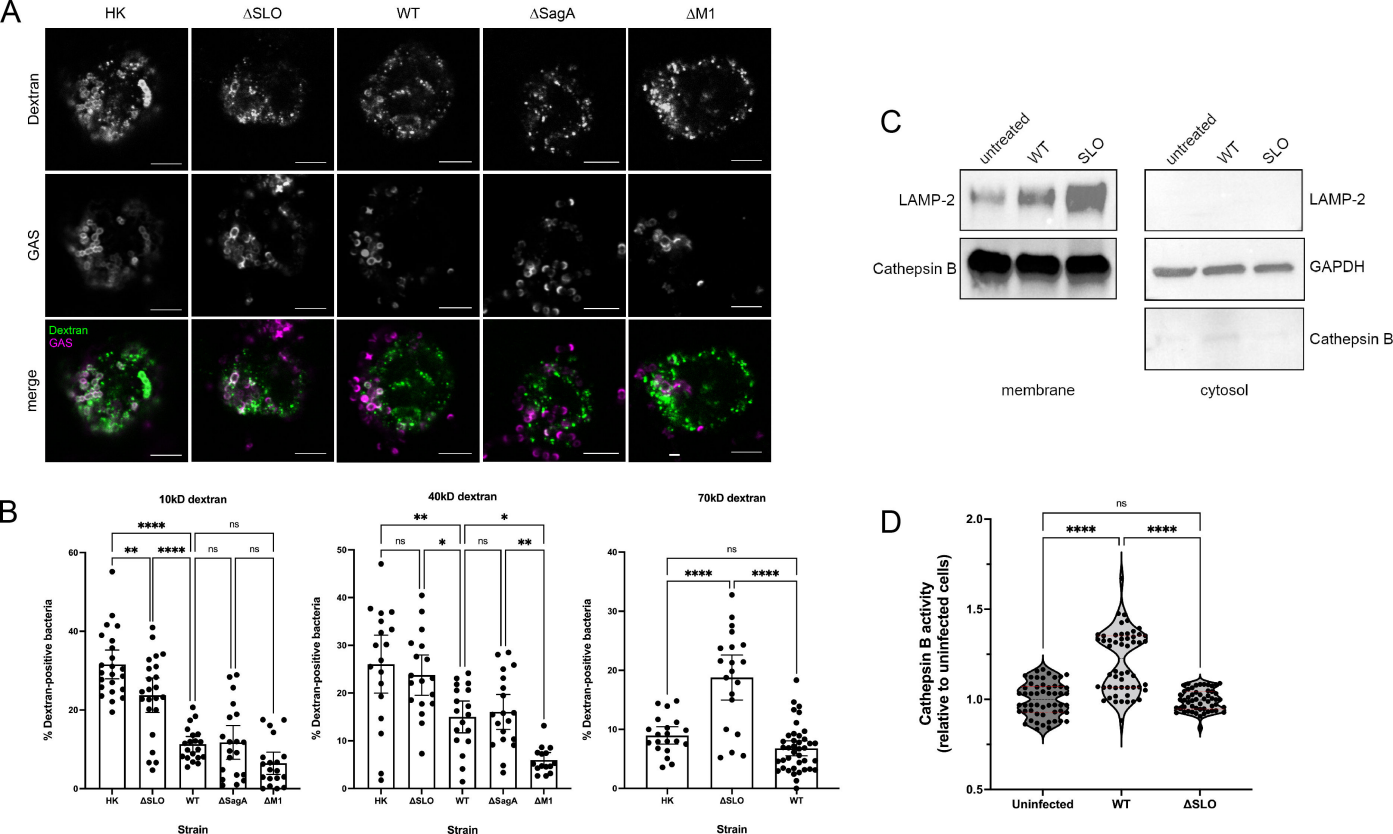
SLO induces phagolysosomal perforation and protein leakage. (**A**) Representative images of the 40-kD dextran probe (top panels, green in merged images) with indicated bacterial strains (middle panels, magenta in merged images). All images were taken with a 63x objective with 2x digital zoom, scale bar = 5 mm. (**B**) Quantitation of colocalization of indicated bacterial strains with 10-, 40-, and 70-kD dextran probes; >100 cells were counted. (**C**) Cells were infected with the indicated bacterial strains, fractionated into membrane (left) and cytosolic (right) fractions, and probed with the indicated antibodies: LAMP-2 (lysosomal marker; loading control for membrane fraction), GAPDH (cytosolic marker; loading control for cytosolic fraction), and cathepsin B (lysosomal enzyme). (**D**) Cathepsin B activity was measured in cytosolic cell fractions collected from cells infected with the indicated strains. Data were normalized to the corresponding protein concentration for each sample and were compared with the uninfected cells. Data in (**B**) and (**D**) were combined from at least three independent experiments. Results are given as mean ± 95% confidence interval (CI), and statistics were performed on arcsin-transformed data by one-way ANOVA with Tukey’s multiple comparison test (**B**) or one-way ANOVA with Kruskal-Wallis multiple comparison test (**D**).

Although GAS has several acid stress response strategies (41), a better tactic may be to avoid the acidification of the phagolysosome altogether. The inability of bacteria to survive in low pH environments (Fig. 3) led us to examine whether GAS infection has a mechanism to prevent phagolysosomal acidification. Pore-forming toxins, such as SLO, create pores as large as 25–30 nm in diameter (16). Others have shown that pore-forming toxins, including SLO, allow escape of hydrogen ions that prevent acidification of the lysosome (15, 42) and proteins such as galectin-8 and galectin-3 to access the interior of the damaged lysosome (15, 43). Streptolysin S (SLS) can similarly perforate membranes (44). We fed THP-1 macrophages fluorescent-conjugated molecules and dextrans of various sizes to assess pore size and leakage from phagolysosomes in GAS-infected cells. In addition to measuring whether leakage occurs, this method has the added benefit of allowing measurement of the size of proteins that could escape from the phagolysosome. The ability to measure phagosomal leakage was confirmed using the lysosomal permeabilization agent LLOMe (Fig. S4). LLOMe permeabilizes lysosomes to molecules up to at least 4.4 kD but not to molecules greater than 10 kD (29). Retention of the 10 kD but not the 570 Da probe in LLOMe-treated cells confirmed uptake of the probe in phagolysosomes and appropriate release into the cytosol upon permeabilization (Fig. S4A). Cells loaded with probes of varying sizes were infected with GAS (Fig. 4A and B). Leakage of the dextran probe was benchmarked to HK bacteria that retain bacterial surface structure but are unable to secrete proteins such as SLO and SLS, and thus are not expected to leak dextrans (Fig. 4A and B). Fluorescent probes were retained and colocalized with heat-killed bacteria, suggesting that a secreted toxin is responsible for leakage (Fig. 4A and B). As expected, WT GAS caused probes up to 40 kD in size, approximately 9 nm in diameter (45), to leak from phagolysosomes (Fig. 4A and B). These data are in agreement with the previous work that galectins that are 30–35 kD can access GAS-infected lysosomes (15, 43). Colocalization of the 70-kD dextran with WT bacteria was similar to HK bacteria, indicating retention of the 70-kD probe (Fig. 4B). To determine which toxins were responsible for this leakage, we infected cells with bacteria lacking different pore-forming toxins. ΔSLO GAS-infected cells retained the probes, while cells infected with ΔSagA GAS (lacking SLS) allowed leakage of the probes, similar to the WT strain (Fig. 4A and B). In our assays, SLS did not significantly allow proteins to escape the lysosome, as indicated by the lack of leakage of even the small 570-Da probe (Fig. S4B). Although these data do not strictly rule out the possibility that SLS could allow proton leakage, they do provide evidence that SLO extensively damages the lysosomal membrane to allow large proteins to escape to the cytosol. Mutant bacteria lacking the surface protein M1 (ΔM1) but with unaltered expression of SLO and SLS behaved similarly to WT strains as expected (Fig. 4A and B). Our data further indicate that SLO allowed probes greater than 40 kD in size, approximately 13 nm in diameter (45), to escape from the lysosomes (Fig. 4B), in agreement with the reported pore size for SLO (16). These data indicate that SLO is the primary pore-forming toxin that perforates the phagolysosome and allows leakage of not only protons but also relatively large proteins.

Because such large molecules can leak from GAS-infected phagosomes, we next tested whether lysosomal enzymes could escape into the cytosol through SLO-mediated pores. We infected cells with WT or ΔSLO GAS and collected membrane and cytosolic cell fractions. Lack of lysosomal markers in the cytosolic fraction indicated little to no contamination of the cytosolic fraction with lysosomes (Fig. 4C). The membrane fraction contained most of the lysosomal enzyme cathepsin B as expected (Fig. 4C). However, we noted a small but visible increase in the amount of cathepsin B present in the cytosolic fraction of WT-infected cells compared with uninfected and ΔSLO-infected cells (Fig. 4C). In order to verify that cathepsin B was present in the cytosol, we measured cathepsin B activity in the cytosolic fraction. Cytosolic fractions from WT-infected cells contained the highest amount of cathepsin B activity compared with uninfected cells (Fig. 4D). Cytosolic fractions from ΔSLO-infected cells (Fig. 4B) did not exhibit cathepsin B activity higher than uninfected cells (Fig. 4D). These data confirmed that SLO secreted by WT bacteria perforates the phagolysosomal membrane and allows the leakage of large proteins, such as the lysosomal enzyme cathepsin B, into the cytosol. This loss of proteolytic enzymes from the phagolysosome in addition to the loss of protons likely contributes to the ability of GAS to persist in the phagolysosome during infection and has implications for the subsequent response of the cell to GAS infection.

### Vacuolar ATPase function in GAS-infected cells

**Fig 5:**
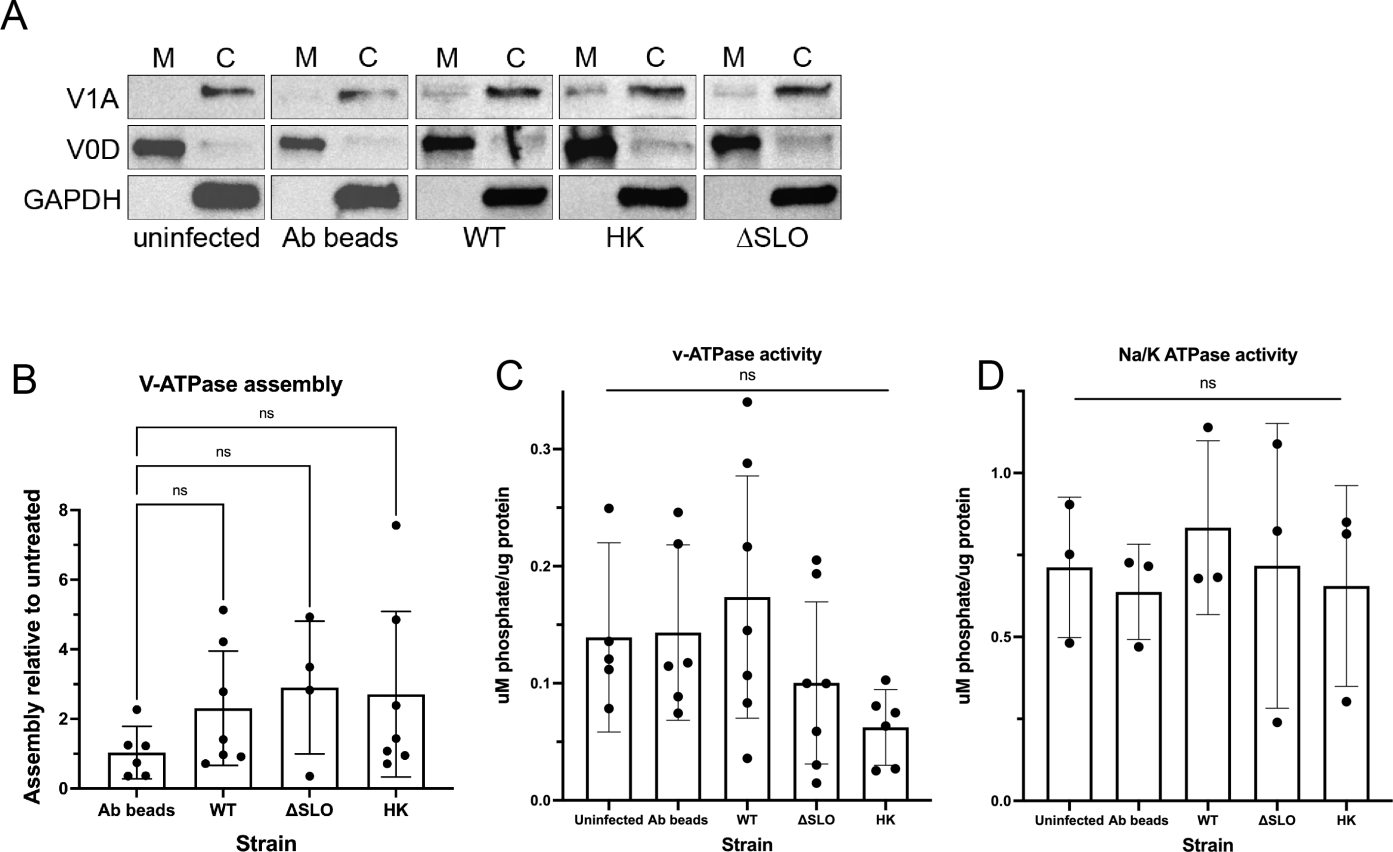
v-ATPase assembly and activity in GAS-infected cells. (**A**) Cells were uninfected and infected with immunoglobulin-conjugated beads (Ab beads, control) and with the indicated GAS strain or HK bacteria, and were fractionated into membrane (M) and cytosolic (C) fractions. The fractions were probed with antibodies to V_1_A, V_0_D, or GAPDH proteins. Representative data are shown. (**B**) Quantitation of data shown in (**A**). V_1_A band intensities were quantified and normalized to V_0_D (membrane) or GAPDH (cytosol). Relative assembly was calculated as the amount of V_1_A in the membrane fraction of the indicated infected sample compared with the uninfected sample for each experiment. (**C**) v-ATPase activity assay in membrane fractions from cells infected with the indicated GAS strain. v-ATPase activity was calculated by determining the concentration of free phosphate (released by the ATPase) of the samples and by subtracting the concentration of free phosphate in samples incubated with bafilomycin (v-ATPase inhibitor) from the samples incubated without bafilomycin. (**D**) Na^+^/K^+^-ATPase activity assay in membrane fractions from cells infected with the indicated GAS strain. Na^+^/K^+^-ATPase activity was calculated by subtracting the concentration of free phosphate in bafilomycin-treated samples incubated with sodium orthovanadate (Na^+^/K^+^-ATPase inhibitor) from samples incubated without orthovanadate. Data in (**B–D**) were combined from at least three independent experiments. Results are given as mean ± 95% confidence interval (CI) and were analyzed by one-way ANOVA with Tukey’s multiple comparison test.

Although SLO-mediated pores could cause leakage of protons from the phagolysosome, reports in the literature also suggest that the v-ATPase that is required for acidification of the lysosome is non-functional in GAS-infected cells (19). Supporting this, we found that the phagolysosomes of macrophages infected with ΔSLO and heat-killed bacteria were also not acidified (Fig. 2G). Furthermore, ΔSLO bacteria are trafficked to phagolysosomes, maintain the fluorescent signal of mWASABI, and are maintained at constant numbers in THP-1 cells similar to WT bacteria (Fig. S5). These data are consistent with the idea that phagolysosomes infected with ΔSLO bacteria are also not acidified. We therefore investigated lysosomal acidification in GAS-infected cells. v-ATPase, the enzyme responsible for lysosomal acidification, consists of the membrane-bound F_0_ subunit (containing the V_0_D_1_ protein) that must assemble with the cytosolic F_1_ subunit (containing the V_1_A protein) for proper function (28). We monitored v-ATPase assembly by tracking movement of the V_1_A subunit from the cytosolic to membrane fractions. Cells fed Ig-conjugated beads (Ab beads) were included as a positive control (Fig. 5A and B). We observed no difference in v-ATPase subunit assembly in cells infected with GAS compared with Ab bead-fed or uninfected cells (Fig. 5A and B), indicating that the acidification machinery was recruited properly to phagolysosomes. We next measured v-ATPase activity in membrane fractions of GAS-infected cells. We found very little v-ATPase activity in our lysosomal fractions, including from cells fed Ab beads as a positive control (Fig. 5C). We could not discern differences in v-ATPase activity between controls and all strains of GAS (Fig. 5C). In contrast, we could readily detect Na^+^/K^+^ ATPase activity (Fig. 5D). Thus, although v-ATPase activity could be altered in infected cells, we could not detect measurable activity.

### GAS in phagolysosomes recruits CD63/LAMP-3

**Fig 6:**
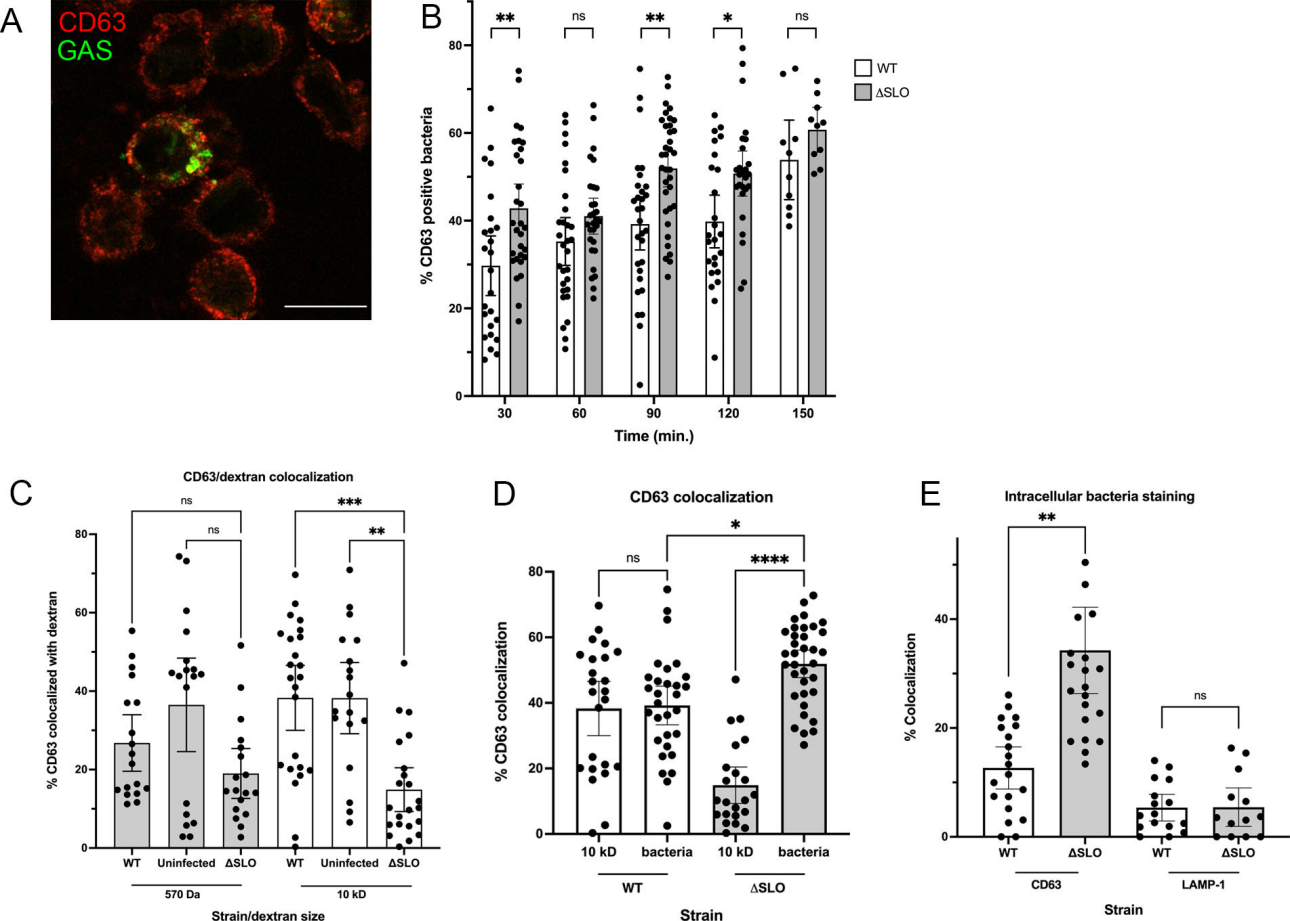
GAS recruitment of CD63 may permeabilize lysosomes in the absence of SLO. (**A**) Representative image of THP-1 cells infected with GAS for 90 min, fixed and probed with anti-CD63 and secondary fluorescent antibodies (red). Bacteria opsonized with human serum were detected with anti-human IgG antibody (green). Scale bar = 5 mm. (**B**) Time course quantitation of bacteria colocalized with CD63. (**C**) Quantitation of 570-Da or 10-kD dextrans colocalized with CD63 in uninfected cells or cells infected with the indicated strains. (**D**) Quantitation of colocalization of CD63 with either 10-kD dextran or the indicated bacterial strain. (**E**) Quantitation of recovered intracellular bacteria stained for either LAMP-1 or CD63. For all figures, data from at least three independent experiments were combined. For (B–E), >100 cells were counted per experiment. Results are given as mean ± 95% confidence interval (CI), and statistics were performed on arcsin-transformed data by one-way ANOVA with Dunnett’s multiple comparison test.

Despite our inability to measure v-ATPase activity, our data suggest that phagolysosomes infected with ΔSLO bacteria are also not acidified (Fig. 2G; Fig. S5). Recent literature suggests that the tetraspanin CD63 (also known as LAMP-3) induces lysosomal permeabilization (46). Because we observed maturation of the phagolysosome via recruitment of LAMP-1 and LAMP-2 (Fig. 1 and 2), we wondered whether CD63 was also recruited to GAS-infected phagolysosomes. A time course analysis of GAS infection by immunofluorescence revealed that GAS associated with CD63 at early time points and that CD63 accumulated with GAS infection over time (Fig. 6A and B). GAS colocalization with CD63 was distinct from colocalization with LAMP-1 (data not shown). Interestingly, a significantly higher proportion of ΔSLO bacteria associated with CD63 compared with the WT strain (Fig. 6B). In a parallel experiment, when dextran-loaded cells were infected with GAS, less CD63 associated with dextran-containing phagosomes when infected with ΔSLO bacteria compared with the WT strain (Fig. 6C). This suggested that CD63 was more highly recruited to phagolysosomes containing ΔSLO bacteria compared with those containing dextran. When we directly compared CD63 colocalization with bacteria or dextrans, CD63 was recruited equally to either bacteria or 10-kD dextran-containing phagolysosomes in cells infected with WT GAS (Fig. 6D). However, in cells infected with ΔSLO GAS, CD63 was more highly recruited to bacteria-containing phagolysosomes compared with the 10-kD dextran-containing phagolysosomes (Fig. 6D). Finally, we performed a staining of recovered intracellular bacteria (Fig. 3C) and found that intracellular ΔSLO GAS was significantly more associated with CD63 compared with WT bacteria and in contrast to LAMP-1 staining (Fig. 6E). Based on these results, we propose that in the absence of SLO, GAS recruits CD63 to permeabilize the lysosomal membrane. This would provide a neutralized pH environment for ΔSLO GAS in phagolysosomes and allow persistence of the bacteria, similar to the WT strain that can achieve this using SLO.

## DISCUSSION

The human-specific pathogen GAS has co-evolved with the human immune system and has multiple mechanisms for survival within the host (1, 11). Although GAS is an extracellular bacterial pathogen, phagocytic cells such as macrophages can readily engulf bacteria (13, 15, 32). The ability of GAS to not only survive in macrophages but also to potentially alter their function, escape downstream immune responses, and be sheltered from antibiotic treatment provides a basis for successful persistence in humans.

In our experiments, we found that GAS was readily phagocytosed and phagosomes quickly fused with lysosomes, but that GAS remained in these compartments (Fig. 1 and 2). There is conflicting evidence in the literature about whether lysosomes fuse with GAS-containing phagosomes in macrophages, which may be due to a difference in cell origin or source of antibodies (13, 15). We find that in THP-1 macrophages, lysosomal fusion with GAS-containing phagosomes occurs, as confirmed by colocalization with three independent lysosomal markers (Fig. 1, 2, and 6). We recognize that although our studies may be limited due to the exclusive use of cell culture, our data are in agreement with several studies regarding lysosomal fusion with GAS-infected phagosomes (15, 32). In addition, we corroborated previous data using an independent method that SLO induces perforation and damage of the phagolysosomal membrane (Fig. 4) (15, 43). Although ΔSLO GAS also prevented phagolysosomal acidification (Fig. 2), others have shown that ΔSLO GAS are less fit for survival in macrophages and other cells (15, 47). ΔSLO GAS is likely still susceptible to other macrophage-killing mechanisms such as reactive oxygen species or xenophagy (47). Finally, our data demonstrate that bacteria persist in phagolysosomes or escape into the cytosol without replicating (Fig. 1 to 3; Fig. S5) (13, 15). This is in agreement with another study of GAS in THP-1 cells (32) but different from what has previously been observed in epithelial and U-937 cells, where GAS escapes into the cytosol and replicates (14, 32, 43). The subsequent fate of GAS in non-functional lysosomes in THP-1 macrophages remains to be elucidated.

The pH of the lysosomal lumen has been reported to range between pH 4.5 and 5 (35). However, we found that acidified medium was sufficient to kill GAS (Fig. 3). These results are somewhat surprising because not only does GAS produce lactic acid, but previous reports also suggest GAS has multiple mechanisms for acid tolerance, including a F_0_/F_1_ proton pump and the arginine deiminase pathway, which produces alkali (36, 37, 41, 48). However, there may be a limit to which these systems work, especially within an enclosed environment such as *in vitro* conditions or in the phagolysosome, as demonstrated by the threshold for bacterial survival in low pH medium (Fig. 3) (37). Additionally, GAS lacks enzymes such as glutamate decarboxylase and urease (41), making neutralization of highly acidified environments a less viable strategy. Therefore, two mechanisms to perforate the phagolysosomal membrane to prevent acidification (Fig. 4 and 6) provide a means to survive within the phagolysosomal compartment. Lysosomal permeabilization by the tetraspanin CD63 is a relatively new finding and the mechanism by which this occurs is not known (46). However, CD63 is also associated with the formation of extracellular vesicles and limits autophagic flux (46, 49). A study with uropathogenic *Escherichia coli* demonstrated that bacteria neutralize lysosomal pH to escape autophagolysosomal digestion and are expelled by exocytosis (50). A similar mechanism could be employed by GAS to facilitate escape from the macrophage. Although we could not sensitively detect v-ATPase activity, others have suggested that GAS also can inhibit v-ATPase activity (19). Other bacteria such as *Legionella pneumophila* have proteins such as SidK, which binds v-ATPase subunits to prevent activity (30). Because our data indicate that HK bacteria also prevent acidification (Fig. 2G), inhibition of v-ATPase activity may be an additional survival strategy GAS employs.

Previous data and our results indicate SLO causes leakage of lysosomal contents (Fig. 4). Lysosomal membrane permeabilization normally triggers autophagic pathways to clear damaged lysosomes (51), but because GAS can inhibit autophagy (52), retaining permeabilized lysosomes may result in more serious consequences, such as releasing active lysosomal enzymes, such as cathepsin B, into the macrophage cytosol (Fig. 4). The cathepsin B activity we measured was subtle, likely because the enzyme optimally functions in a low pH environment (7), which is neither achieved in GAS-infected lysosomes (Fig. 2 and 5) nor in the cytosol. However, releasing active lysosomal proteases could have downstream effects on the cell such as activation of the NLRP3 inflammasome and pyroptosis (53). Furthermore, SLO promotes macrophage apoptosis by releasing SLO from the lysosome and causing mitochondrial damage (21, 54). Other proteins released from the infected lysosome could also include bacterial proteases such as SpeB, whose presence in the cytosol could also lead to NLRP3 inflammasome activation and release of the proinflammatory cytokine IL-1ꞵ (55–59). Persistent IL-1ꞵ activation as a result of GAS infection has been linked with ARF and rheumatic heart disease (60). Thus, besides enabling bacterial survival and blunting the normal macrophage response, this alternative macrophage response may lead to pathologies, such as ARF, which are observed during GAS infection. Studies have shown that macrophages are crucial for GAS clearance (2), and the loss of macrophage function may also have detrimental effects on the long-term response to GAS infection, including the inability to generate protective antibodies. Therapies aimed at improving macrophage function may therefore improve GAS infection outcomes. Although SLO is a promising target, our work shows that additional drugs to restore lysosomal integrity and function would be necessary to fully restore macrophage function. Restoration of macrophage function in GAS infection would allow individuals to mount a proper adaptive immune response to confer protective immunity.

## SUPPLEMENTAL MATERIAL

Fig. S1 iai.00141-24-s0001.tif Percentage of cells infected with GAS.

Fig. S2 iai.00141-24-s0002.tif Lysotracker detects GAS.

Fig. S3 iai.00141-24-s0003.tiff Short-term GAS infection does not produce significant toxicity.

Fig. S4 iai.00141-24-s0004.tif Fluorescent probes appropriately monitor phagolysosomal leakage.

Fig. S5 iai.00141-24-s0005.tif ΔSLO GAS persists in THP-1 phagolysosomes.

Supplemental material iai.00141-24-s0006.docx Supplemental methods; legends for Fig. S1 to S5.

ASM does not own the copyrights to Supplemental Material that may be linked to, or accessed through, an article. The authors have granted ASM a non-exclusive, world-wide license to publish the Supplemental Material files. Please contact the corresponding author directly for reuse.
