# Supplementary figures and images for: Group A Streptococcus induces lysosomal dysfunction in THP-1 macrophages
Source: Infect Immun. 2024 May 9;92(6):e00141-24. doi: 10.1128/iai.00141-24 (PMC11237432; doi:10.1128/iai.00141-24)

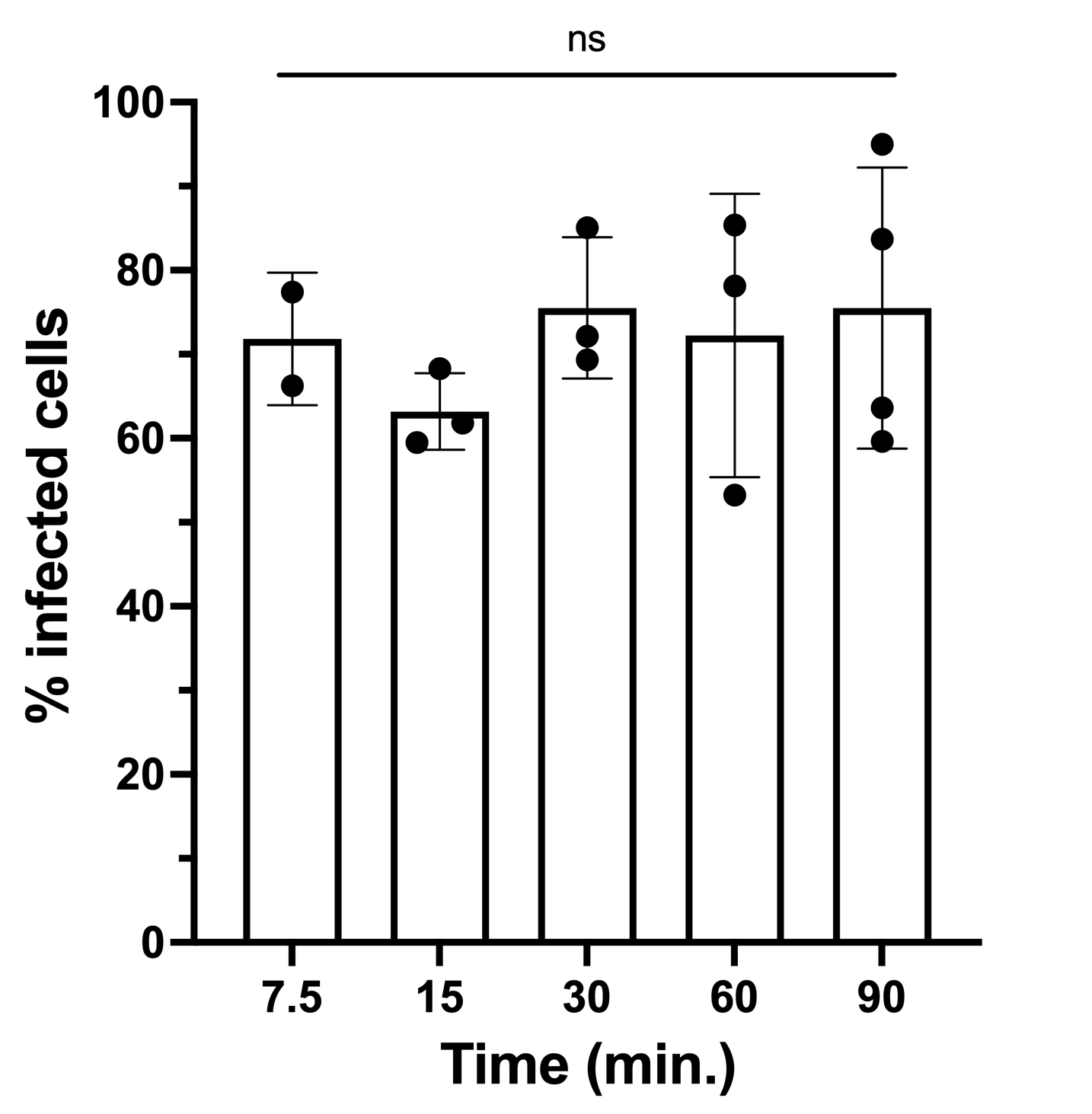

Supplement: Fig. S1 — Percentage of cells infected with GAS. [file iai.00141-24-s0001.tif]

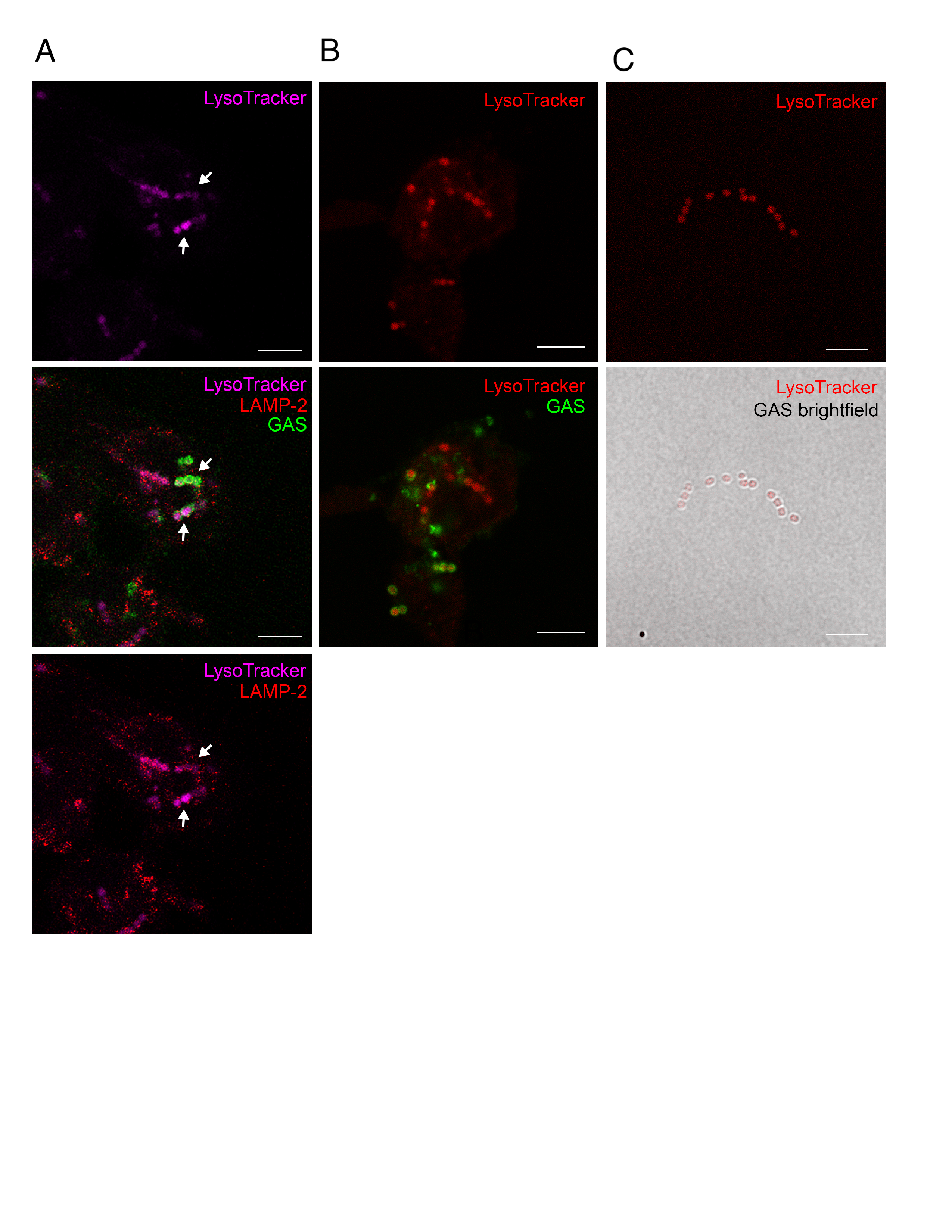

Supplement: Fig. S2 — Lysotracker detects GAS. [file iai.00141-24-s0002.tif]

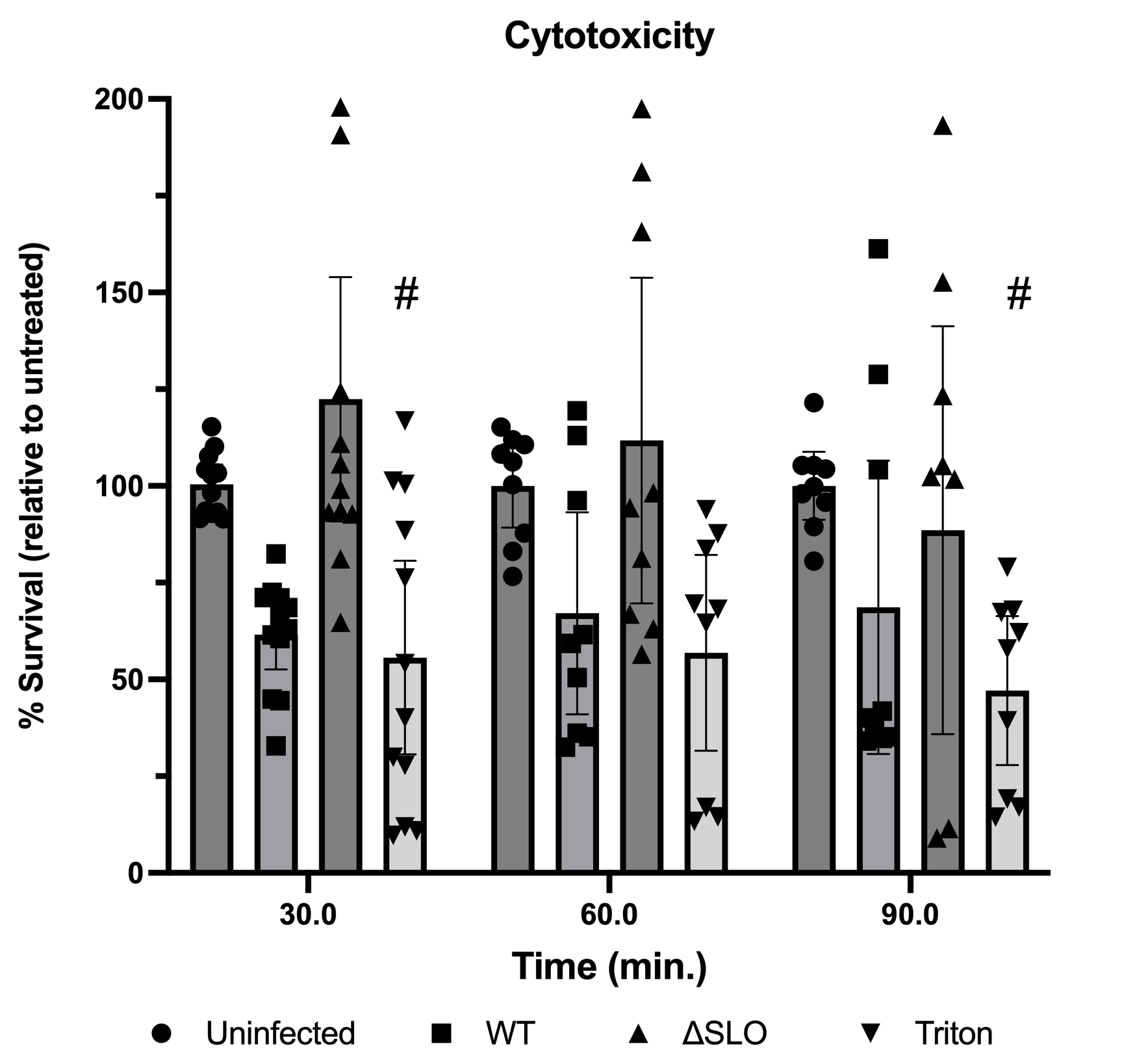

Supplement: Fig. S3 — Short-term GAS infection does not produce significant toxicity. [file iai.00141-24-s0003.tiff]

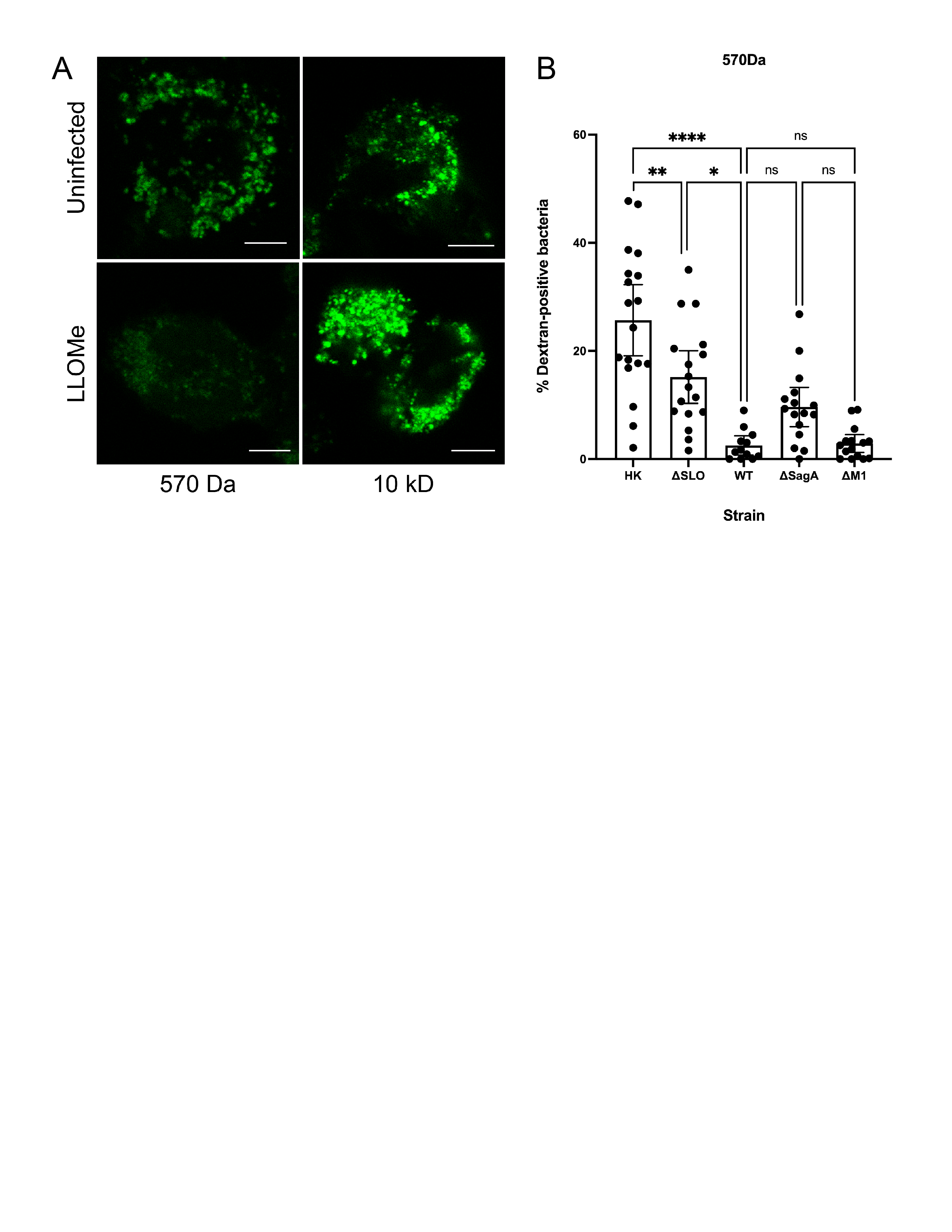

Supplement: Fig. S4 — Fluorescent probes appropriately monitor phagolysosomal leakage. [file iai.00141-24-s0004.tif]

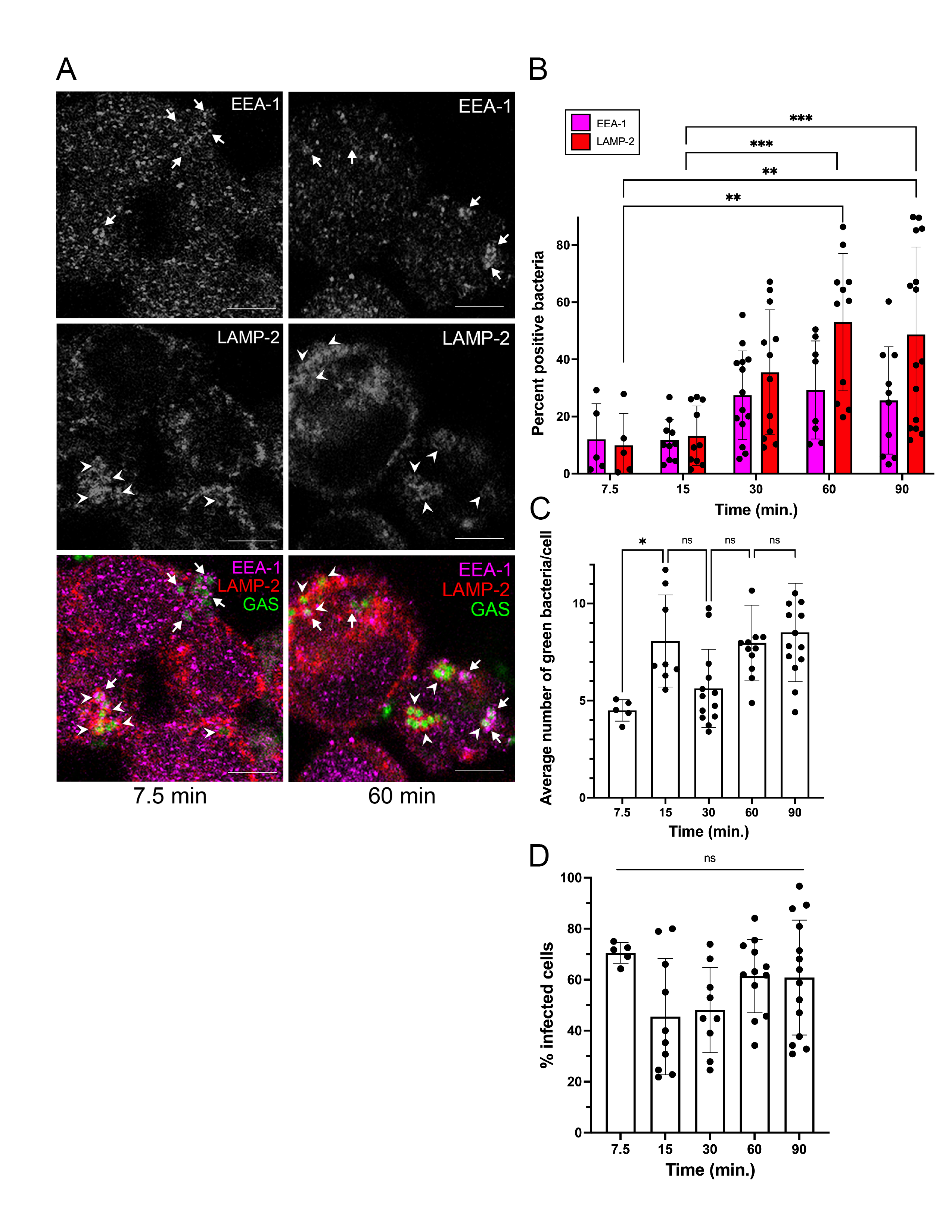

Supplement: Fig. S5 — ΔSLO GAS persists in THP-1 phagolysosomes. [file iai.00141-24-s0005.tif]
